# Pro-Resolving Effects of Resolvin D_2_ in LTD_4_ and TNF-α Pre-Treated Human Bronchi

**DOI:** 10.1371/journal.pone.0167058

**Published:** 2016-12-09

**Authors:** Rayan Khaddaj-Mallat, Chantal Sirois, Marco Sirois, Edmond Rizcallah, Sofia Marouan, Caroline Morin, Éric Rousseau

**Affiliations:** 1 Department of Obstetrics-Gynecology Faculty of Medicine and Health Sciences, Université de Sherbrooke, Sherbrooke, Quebec, Canada; 2 Service of Thoracic Surgery, CHUS Felurimont, Sherbrooke, Quebec, Canada; 3 Department of Pathology, Faculty of Medicine and Health Sciences, Université de Sherbrooke, Sherbrooke, Quebec, Canada; 4 Nursery School, Université de Montréal, Montreal, Quebec, Canada; Duke University School of Medicine, UNITED STATES

## Abstract

Inflammation is a major burden in respiratory diseases, resulting in airway hyperresponsiveness. Our hypothesis is that resolution of inflammation may represent a long-term solution in preventing human bronchial dysfunctions. The aim of the present study was to assess the anti-inflammatory effects of RvD_2_, a member of the D-series resolving family, with concomitant effects on ASM mechanical reactivity. The role and mode of action of RvD_2_ were assessed in an *in vitro* model of human bronchi under pro-inflammatory conditions, induced either by 1 μM LTD_4_ or 10 ng/ml TNF-α pre-treatment for 48h. TNF-α and LTD_4_ both induced hyperreactivity in response to pharmacological stimuli. Enhanced 5-Lipoxygenase (5-LOX) and cysteinyl leukotriene receptor 1 (CysLTR1) detection was documented in LTD_4_ or TNF-α pre-treated human bronchi when compared to control (untreated) human bronchi. In contrast, RvD_2_ treatments reversed 5-LOX/β-actin and CysLTR1/β-actin ratios and decreased the phosphorylation levels of AP-1 subunits (c-Fos, c-Jun) and p38-MAP kinase, while increasing the detection of the ALX/FPR2 receptor. Moreover, various pharmacological agents revealed the blunting effects of RvD_2_ on LTD_4_ or TNF-α induced hyper-responsiveness. Combined treatment with 300 nM RvD_2_ and 1 μM WRW4 (an ALX/FPR2 receptor inhibitor) blunted the pro-resolving and broncho-modulatory effects of RvD_2_. The present data provide new evidence regarding the role of RvD_2_ in a human model of airway inflammation and hyperrresponsiveness.

## Introduction

In chronic airway diseases, the distal parts of the bronchial tree have been recognised as a predominant site of airway inflammation and hyperrresponsiveness, due to the production of cytokines (TNF-α, IL-13, etc.) and eicosanoids (LTD_4_, PAF, PG) involved in the inflammatory process [1, 2]. Distal inflammation has moreover been described as more severe when compared to large airway inflammation, with emerging evidence of peripheral lung remodelling [3].

Among the inflammatory mediators that are synthesised by inflamed airways, the highly potent leukotrienes, derived from the 5-lipoxygenase (5-LOX) pathway of arachidonic acid metabolism, have been recognised as powerful spasmogens of airway smooth muscle (ASM) cells [2, 4, 5]. Evidences for LTD_4_ as mediators of inflammation as well as a bronchoconstrictor are well demonstrated [6, 7, 8]. Initially, LTD_4_ promotes inflammation and induces both Ca^2+^ flux and extracellular signal-regulated kinase activation, leading to airway hyper-responsiveness [6, 8]. In addition, LTD_4_ is recognised for its potent bronchoconstrictive activity, this eicosanoid binds CysLTR1 receptors that are highly expressed in the respiratory tract [6, 7].

In parallel, numerous pro-inflammatory cytokines such as TNF-α are involved in chronic airway diseases for their underlying role in inflammatory events, including the production of leukotrienes [9, 10]. Thus, pharmacological agents that can either suppress the production of TNF-α or block its biological actions may have potential therapeutic value in airway inflammatory diseases [11, 12].

Distinct studies have reported the benefits of n-3 polyunsaturated fatty acids (n-3 PUFAs) in maintaining human health due to the endogenous metabolism and production of multiple specialised pro-resolving mediators, such as resolvins (RvDs and RvEs), protectins (PDs) and maresins (MaRs) [13, 14, 15, 16, 17]. Concentrations of the D-series Resolvins within the biological range were shown to induce anti-inflammatory and pro-resolving activities in isolated human leucocytes *in vitro* and in a mouse model of acute inflammation *in vivo* [18]. In chronic respiratory diseases, RvDs have been identified as important pro-resolving agents by blunting airway inflammatory markers [15, 19]. Among n-3 PUFAs, docosahexaenoic acid (DHA) increases the production of these anti-phlogistic mediators over time during the course of the inflammatory process [15]. For example, RvD_1_ and RvD_2_ have long been associated with pro-resolving effects in chronic diseases [17, 20]. RvD_1_ blunts IL-13-induced ASM inflammation [21] and reverses the nuclear localisation of 5-LOX in macrophages [22], while RvD_2_ treatment inhibits mouse aortic smooth muscle cell migration by blunting TNF-α-stimulated p65 translocation [23]. Moreover, RvD_2_ has been reported to curb neutrophil inflammation and endothelial cell adhesion in a mouse burn wound and healing model [24] as well as microbial sepsis in mice [25].

Our working hypothesis is that LTD_4_ or TNF-α induces airway hyperresponsiveness in human distal bronchi (0.5–0.8 mm diameter) and that RvD_2_ treatment inhibits the main inflammatory biomarkers in distal airways which in turn, would result in lower bronchial reactivity. Hence, RvD_2_ could represent a potential compound to alleviate this hyperresponsiveness. The aim of the present study was therefore to assess the effects of RvD_2_ on bronchial inflammatory markers and pharmacologically-induced tone using human bronchi. Herein, we report the first evidence that RvD_2_ displays resolving properties and prevents airway hyperresponsiveness.

## Materials and Methods

### Collection of lung resection samples

This study was approved by the institutional Ethics Committee of the *Centre Hospitalier Universitaire de Sherbrooke* (protocol number: 05 088 S2-M2) and was designed in collaboration with the Service of Thoracic Surgery and the Department of Pathology. After providing the written informed consent, human lung tissues were obtained from 16 patients (N = 16) undergoing lobectomy for adenocarcinoma resection. The number of retrieved human bronchi (HB) differed depending on the size of the lung resection (n = 10–12 bronchi / human lung resection). Following the pathological analysis, the absence of carcinoma infiltration was retrospectively established in all lung tissues [21, 19].

### Isolation and culture of human distal bronchi

Tissue samples were placed in Krebs solution, pH 7.4, at 22°C and immediately transported to a level 2-culture room. After removal of connective tissue and adhering parenchyma, paired rings of similar weight and length (inner diameter of 0.5–0.8 mm) were microdissected. Bronchial rings were placed in individual wells of 24-well culture plates as described in ref 21, 35. Human bronchi were either untreated (control) or treated with 10 ng/ml TNF-α, TNF-α + 300 nM RvD_2_, TNF-α + RvD_2_ + 300 nM WRW4 (ALX/FPR2 receptor inhibitor) or TNF-α + RvD_2_ + 1 μM WRW4. Other series of human bronchi were pre-treated with 1 μM LTD_4_ or 1 μM LTD_4_ + 300 nM RvD_2_. All tissues were incubated at 37°C in 5% CO_2_. Note that the use of 300 nM RvD_2_ was based on previous publications [21, 26].

### Mechanical tension measurements

Tension measurements were performed using an isolated organ bath system (Radnoti Glass Tech., Monrovia, CA) as previously described [21]. Passive and active tensions were assessed using FT03 Grass transducer systems coupled to Polyview software (Grass-Astro-Med Inc, West Warwick, RI) for data acquisition and analysis [21, 19].

### Preparation of bronchial homogenates

Human bronchi were weighed and promptly transferred in a buffer containing (mM): 300 sucrose, 20 K-PIPES, 4 K-EGTA, pH 7.2 and a cocktail of protease and phosphatase inhibitors (protease-inhibitor pellets from Roche Diagnostics, Indianapolis, IN, USA, plus 10 μM Na_2_VO_3_). Tissues were homogenised on ice, frozen in liquid nitrogen, and stored at—80°C [21, 19].

### SDS-PAGE and western blot analyses

Western blots were performed on human bronchial homogenates using specific antibodies against ALX/FPR2, CysLTR1, 5-LOX, P-c-Fos, c-Fos, P-c-Jun, c-Jun, P-p38-MAPK, p38-MAPK and β-actin proteins. Blot immunostainings were revealed on Kodak film, digitised using a Xerox GPD PS V3.4377.6.0 set at 600 dpi and analysed using ImageJ software [21, 19].

### Data and statistical analyses

Results are expressed as means ± SEM with n indicating the number of experiments. Statistical analyses were performed using a Student *t* test or one-way analysis of variance (ANOVA). Differences were considered statistically significant when *P < 0.05. All statistical analyses were performed with Sigma Plot 12.0 (SPSS-Science, Chicago, IL).

### Drugs and chemical reagents

TNF-α, methacholine chloride (MCh), histamine and β-actin antibodies were purchased from Sigma (St. Louis, MO, USA). RvD_2_, LTD_4_, U-46619, as well as TNF-α and COX-2 antibodies were obtained from Cayman Chemical (Ann Arbor, Michigan). 5-LOX, CysLTR1, P-c-Fos, P-c-Jun, c-Fos, c-Jun, P-p38-MAPK, p38-MAPK antibodies were purchased from Cell Signalling Technology (Boston, MA, USA). DMEM/F-12 and penicillin-streptomycin were obtained from GIBCO Invitrogen Corp. (Burlington, ON, Canada). WRW4 was purchased from Tocris Bioscience (Minneapolis, MN, USA).

## Results

### Effects of RvD_2_ on LTD_4_-induced bronchoconstriction and CysLTR1 expression

**Fig 1:**
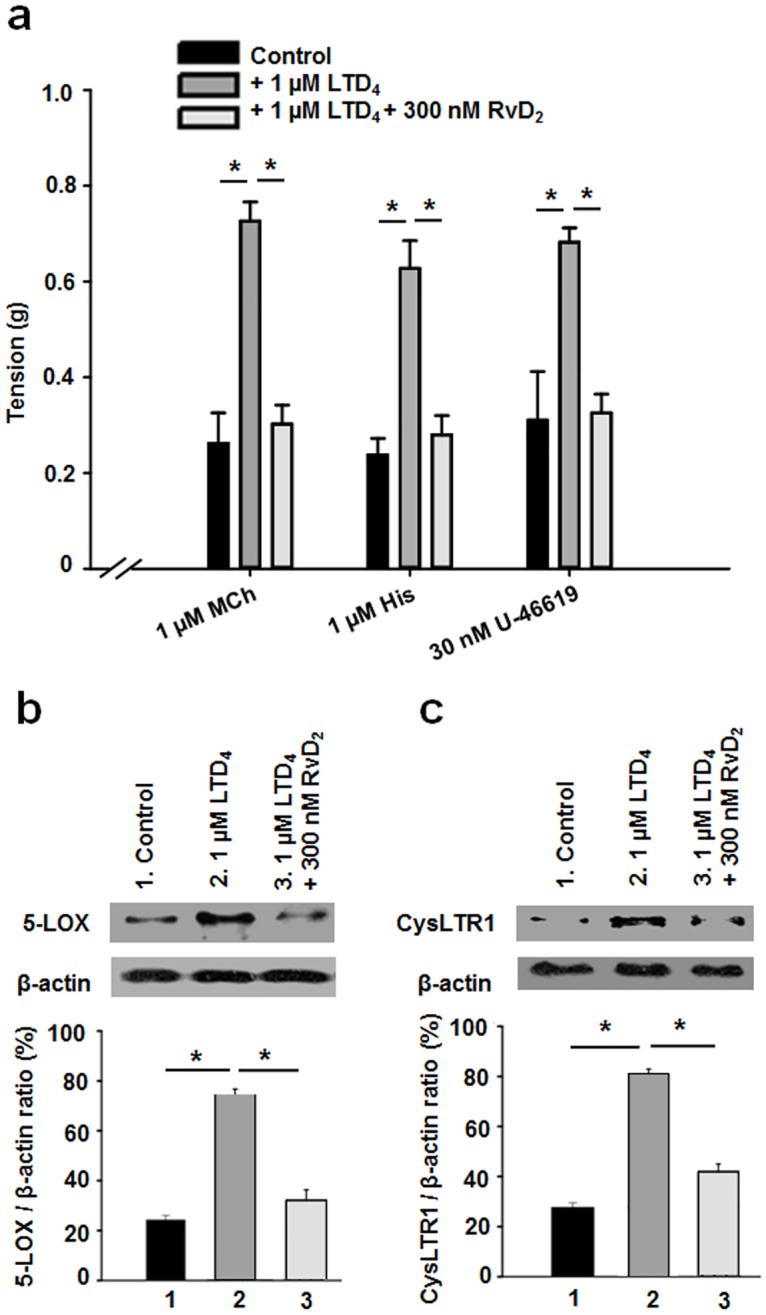
Effect of RvD_2_ on LTD_4_–pretreated human bronchi in response to pharmacologically-induced tone and 5-LOX/CysLTR1 expression. **a.** Corresponding bar graph showing mean contractile amplitudes induced by 1 μM methacholine (MCh), 1 μM histamine or 30 nM U-46619, either under control (untreated) conditions or after 1 μM LTD_4_ pre-treatment in the absence or presence of 300 nM RvD_2_ (n = 7–22, *P < 0.05). **b.** Western blot analyses of the 5-LOX / β-actin ratio assessing the putative effects of 1 μM LTD_4_ and 1 μM LTD_4_ + 300 nM RvD_2_ on human bronchial homogenates compared with control conditions (*n* = 6, *P < 0.05). **c.** Western blot analyses using CysLTR1 and β-actin antibodies. Densities of the immunoreactive bands are expressed as a function of the β-actin-immunoreactive band (*n* = 6, * *P* < 0.05).

To investigate the pharmaco-mechanical properties of LTD_4_-pre-treated human bronchi in the absence or presence of RvD_2_, human bronchi were challenged *in vitro* with a range of relevant pharmacological agonists that included 1 μM MCh (Methacholine), 1 μM His (Histamine) and 30 nM U-46619 (a thromboxane receptor agonist), all of which induced rapid increases in mean mechanical tension. In unsupplemented media, the mean tension of control human bronchi for 1 μM MCh, 1 μM histamine or 30 nM U-46619 was 0.26 ± 0.06 g, 0.23 ± 0.03 g and 0.31 ± 0.10 g, respectively (Fig 1a). Based on CysLTR1/β-actin ratio quantification (see S1 Plate), 1 μM LTD_4_ was deemed as the optimal concentration to treat human distal bronchi in the present work. At this concentration, LTD_4_ increased bronchial contractile responses to the above agents to 0.72 ± 0.04 g, 0.62 ± 0.05 g and 0.68 ± 0.03 g, respectively, compared with control bronchi (Fig 1a). Exposure to 300 nM RvD_2_ markedly reversed the LTD_4_–induced increases in mean tension of challenged bronchi to 0.30 ± 0.04 g, 0.27 ± 0.04 g, and 0.32 ± 0.03 g for MCh, His and U-46619-1, respectively, representing significant decreases in active tension of 58.3%, 56.5% and 53.0%, respectively compared to LTD_4_-pretreated bronchi (Fig 1a). These data reveal that submicromolar concentrations of RvD_2_ are able to prevent LTD_4_-induced hyperreactivity in human bronchi *in vitro*.

The activation of signalling pathways involved in the LTD_4_-induced inflammation process is relatively complex and encompasses various proteins and receptors [6, 8, 11 27]. Among these, 5-LOX is known to be activated prior to its translocation to the nuclear membrane, thus favouring the biosynthesis of LTD_4_. Fig 1b demonstrates that, compared with control conditions (untreated), the expression level of 5-LOX was increased in bronchial homogenates derived from 48-h LTD_4_-pretreated human bronchi, whereas 300 nM RvD_2_ abolished this increase in normalised 5-LOX/β-actin ratio.

One of the most relevant pro-inflammatory effectors of LTD_4_ is the CysLTR1 receptor [27, 28]. The expression of CysLTR1 was detected in human bronchial homogenates and expressed as a function of total β-actin (Fig 1c). Upon pro-inflammatory treatment with 1μM LTD_4_, the relative detection level of CysLTR1 was significantly increased. However, 300 nM RvD_2_ pre-treatment abolished the increase in the CysLTR1/β-actin density ratio (Fig 1c).

### Pro-resolving effect of RvD_2_ on TNF-α-pre-treated human bronchi

**Fig 2:**
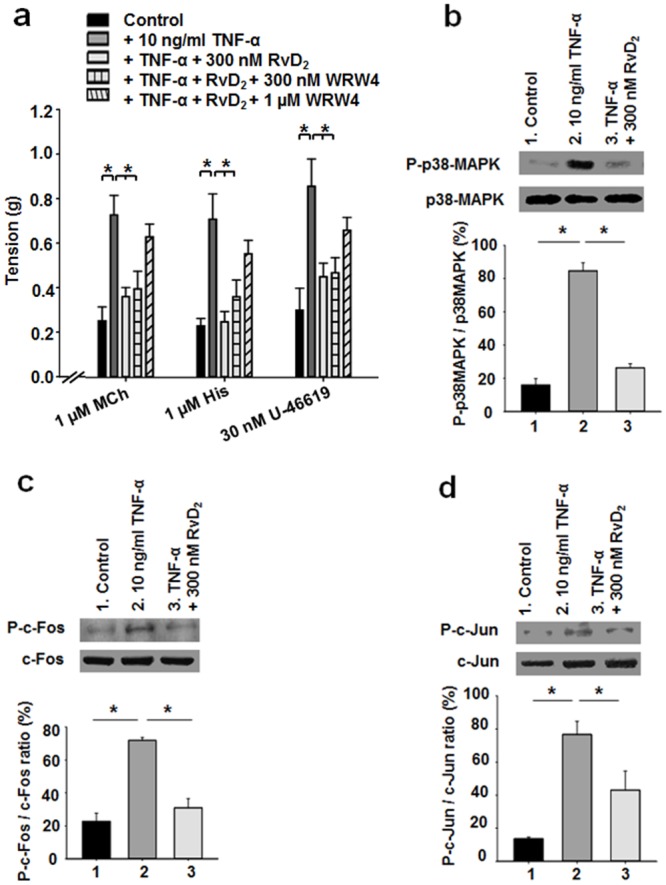
Effect of RvD_2_ on TNF-α-induced reactivity and inflammation in human bronchi. **a.** Bar graph of the contractile activity induced by bronchoactive agents (MCh, His and U-46619) on 48-h cultured human bronchi in control (untreated, n = 7–12) conditions, 10 ng/ml TNF-α (n = 14–19), TNF-α + 300 nM RvD_2_ (n = 15–26), TNF-α + RvD_2_ + 300 nM WRW4 (n = 11–14) or TNF-α + RvD_2_ + 1 μM WRW4 (n = 10–11), *P < 0.05. **b.** Western blot analyses using specific antibodies against P-p38-MAPK and total p38-MAPK. Staining densities of P-p38-MAPK are expressed as a function of p38-MAPK levels (n = 5, * *P* < 0.05). **c.** Western blot analyses were performed using antibodies against p-c-Fos and total c-Fos. The relative density ratio of p-c-Fos to total c-Fos was used to quantify the comparative effects of RvD_2_ on TNF-α-pretreated human bronchi (n = 5, * *P* < 0.05). **d.** Western blot analyses were performed using antibodies against p-c-Jun and total c-Jun. Staining densities of p-c-Jun are expressed as a function of c-Jun levels (n = 5, * *P* < 0.05).

To assess the putative pro-resolving effect of RvD_2_ on pre-established chronic inflammation induced by TNF-α, a series of mechanical tension measurements were performed using various spasmogens. TNF-α pre-treatment consistently increased the reactivity to all bronchoactive agents when compared to controls, whereas 300 nM RvD_2_ treatments largely prevented this TNF-α induced hyperreactivity (Fig 2a).

The activation of signalling pathways involved in airway inflammation is mediated by various nuclear factors [29, 30, 31]. Among these, AP-1 and p38-MAP Kinase, which are under the control of TNF-α receptors, are known to be phosphorylated prior to their translocation into the nucleus, where they activate the transcription of several genes participating in airway inflammation [10, 30, 32, 33]. Fig 2c and 2d demonstrate that, compared to untreated controls, the phosphorylation levels of c-Fos and c-Jun were increased in bronchial homogenates derived from human bronchi treated for 48h with 10 ng/ml TNF-α. In contrast, 300 nM RvD_2_ abolished the increase in normalised P-c-Fos/c-Fos and P-c-Jun /c-Jun ratios (Fig 2c and 2d).

To determine the impact of RvD_2_ on p38 MAPK signalling pathways which control cellular responses to inflammatory cytokines including TNF-α [34], protein levels of phospho-p38-MAPK (P-p38-MAPK) and total p38-MAPK were analysed by Western blots in TNFα-treated human bronchial homogenates. TNFα treatment resulted in an increase in P-p38-MAPK/p38-MAPK density ratio when compared to the mean level obtained in control bronchi. In contrast, RvD_2_ treatments decreased the phosphorylation level of p38-MAPK in lung tissues comparatively to the levels observed in TNFα-treated tissues only (Fig 2b).

### Effects of RvD_2_ and WRW4 on TNF-α-induced airway hyperresponsiveness

In order to determine the impact of RvD_2_ alone or in combination with WRW4 (a specific blocker peptide of the ALX/FPR2 receptor) on airway hyperresponsiveness triggered by TNF-α, various pre-treatments were assessed on the pharmaco-mechanical responses in human bronchial explants. TNF-α treated bronchi displayed a significant over-reactivity to agonist-triggered mechanical responses to all tested pharmacological agents. Conversely, RvD_2_ was able to normalise the increased broncho-reactivity induced by TNF-α in the presence of spasmogens. Moreover, 1 μM WRW4 in combination with 300 nM RvD_2_ displayed broad inhibitory effects than 300 nM RvD_2_ + 300 nM WRW4 in TNF-α-pre-treated human bronchi (see Fig 2a).

### Immunodetection of the ALX/FPR2 receptor in TNF-α-pretreated human bronchi

**Fig 3:**
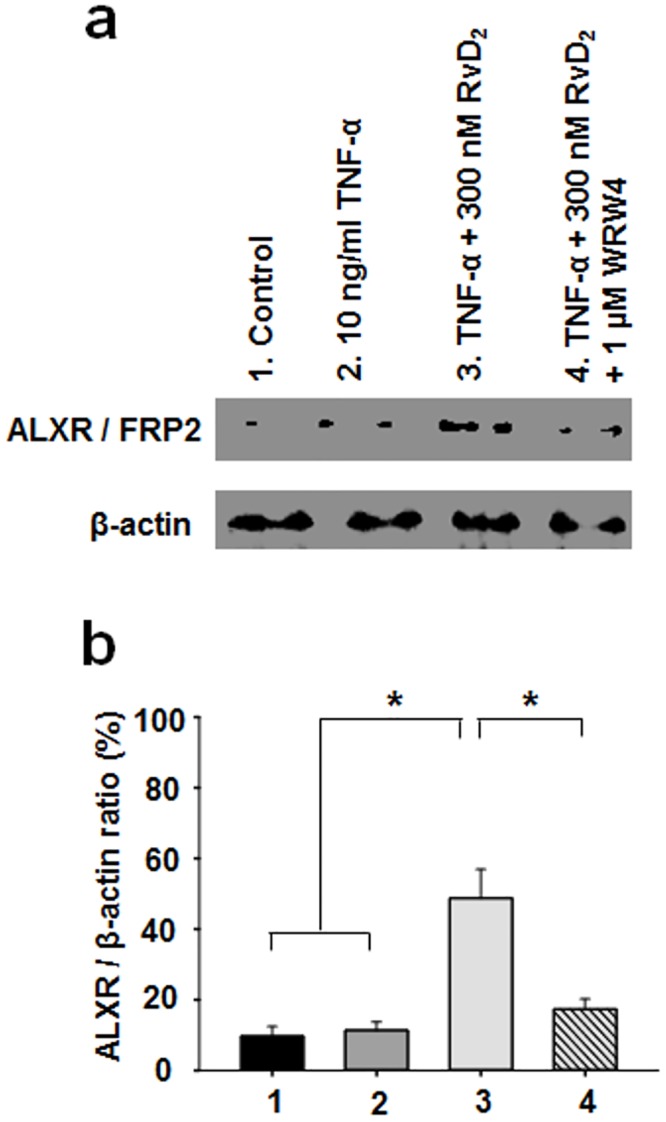
Pharmacological effects of RvD_2_ on ALX / FRP2 protein expression in TNF-α-pretreated human bronchi. **a.** Human bronchial homogenates derived from control (untreated) bronchi or pre-treated with 10 ng/ml TNF-α, TNF-α + 300 nM RvD_2_ or TNF-α + RvD_2_ + 1 μM WRW4 were stained using specific antibodies against ALX / FRP2 and β-actin. **b.** Quantitative analyses of ALX / FRP2 density ratio. Staining densities of ALX / FRP2 are expressed as a function of β-actin staining level (n = 5, * *P* < 0.05).

To test whether the effect of RvD_2_ was mediated through the ALX/FPR2 receptor, 1 μM WRW4 in combination with RvD_2_ treatments was used to assess ALX/FPR2 expression levels in human bronchial homogenates under various experimental conditions. Western blot and quantitative analysis of immunoblots revealed that 48-h treatment with TNF-α did not significantly modulate the expression of ALX/FPR2. However, 300 nM RvD_2_ significantly enhanced the detection of ALX/FPR2 receptors (Fig 3a and 3b), whereas the addition of the WRW4 peptide blocker in combination with RvD_2_ resulted in a loss of ALX/FPR2 receptor density ratio, bringing new light regarding the mode of action of RvD_2_ on ALX/FPR2 protein expression.

### Effect of RvD_2_ and WRW4 on TNF-α-induced 5-LOX / CysLTR1 expression

**Fig 4:**
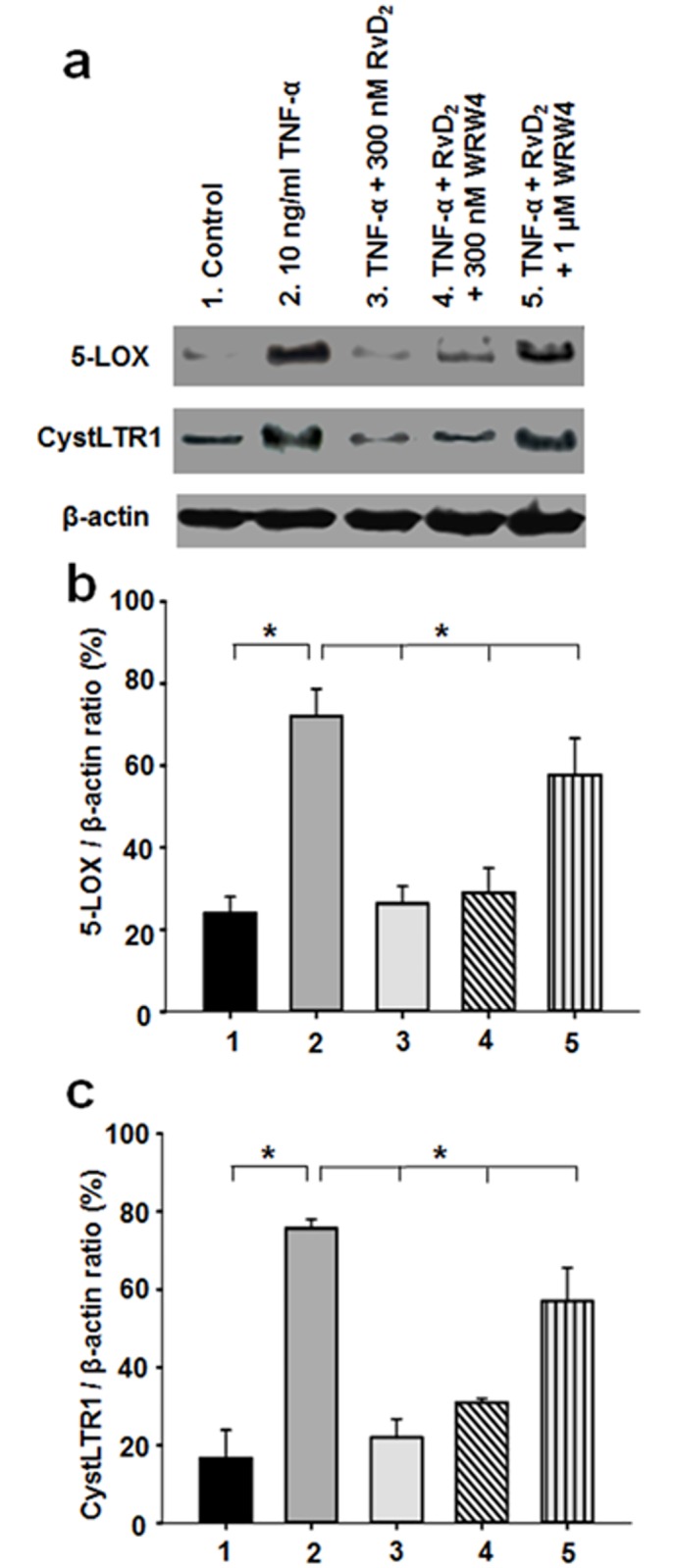
Effect of RvD_2_ and WRW4 treatments on 5-LOX and CysLTR1 expression in TNF-α-pre-treated human bronchi. **a.** Western blot analyses of bronchial homogenates derived from control, TNF-α, TNF-α + 300 nM RvD_2_, TNF-α + 300 nM RvD_2_ + 300 nM WRW4 and TNF-α + RvD_2_ + 1 μM WRW4-pretreated bronchial rings for 48h, using specific antibodies against 5-LOX, CysLTR1 and β-actin, respectively. **b.** Quantitative analysis of various 5-LOX / β-actin density ratios (n = 5, * *P* < 0.05). **c.** Bar graph of mean CysLTR1 / β-actin density ratios in human bronchial homogenates (n *=* 5, * *P* < 0.05).

TNF-α has been demonstrated to stimulate the activity of 5-LOX pathways leading to the synthesis of leukotrienes [9]. To assess whether RvD_2_ blunts TNF-α-induced pro-inflammatory conditions, quantitative analysis demonstrated that, compared to untreated controls, expression levels of 5-LOX/β-actin (Fig 4a and 4b) and CysLTR1/β-actin (Fig 4a–4c) density ratios were increased in human bronchial homogenates treated for 48h with 10 ng/ml TNF-α. In contrast, 300 nM RvD_2_ abolished the above increases in 5-LOX/β-actin and CysLTR1/β-actin ratios. Conversely, the presence of the specific peptide blocker WRW4 eradicated the beneficial effects of RvD_2_ ligands on blunting TNF-α-induced 5-LOX/CysLTR1 activation, resulting in a complete loss of their apparent resolving properties on 5-LOX/β-actin and CysLTR1/β-actin ratios (Fig 4a–4c).

## Discussion

The present study investigated the ability of Resolvin D_2_ (RvD_2_) to prevent the abnormal increase in airway inflammation and pharmaco-mechanical reactivity induced by LTD_4_ or TNF-α mimicking *in vitro* pro-inflammatory conditions in short-term cultured human bronchi. The major findings of this study include (i) the resolving mode of action of RvD_2_ on complementary membrane and cellular inflammatory biomarkers such as CysLTR1, 5-LOX, p38-MAPK and AP-1; (ii) the broncho-modulatory role of RvD_2_ on LTD_4_- and TNF-α-induced airway hyperresponsiveness. We also demonstrate that WRW4, a specific peptide blocker of the ALX/FRP2 receptor, reverses the antiphlogistic and bronchomodulatory effects of RvD_2_, a trihydroxylated DHA metabolite, suggesting a putative role for the ALX/FPR2 receptor in mediating pro-resolving effects of RvD_2_ in human bronchi.

It is well established that the production of leukotrienes is increased in the lungs of asthmatic patients [31, 28], with leucocytes and airway smooth muscle cells shown to be direct and/or indirect sources of these lipid mediators [6, 8, 7, 32]. The role of leukotrienes has been summarised in recent studies and includes: recruitment of airway inflammatory cells, bronchoconstriction and increased vascular permeability [4, 31]. Moreover, there is accumulating evidence that CysLTs may play a role in the remodelling process in chronic asthma, which also includes ASM inflammation and hyperrresponsiveness [8, 2, 31, 28]. Moreover, 5-LOX is required for the first step of leukotrienes synthesis. Indeed, LTD_4_ is primarily synthesised in neutrophils and macrophages and is highly implicated in asthma pathology [28, 33].

On the other hand, n-3 PUFA derivatives have been shown to display pro-resolving effects in various chronic airway diseases by stimulating the clearance of inflammatory debris and promoting mucosal antimicrobial defence [15, 16]. To date, no study has described the antiphlogistic role of D series Resolvins in leukotrienes-induced airway inflammation. Previous mechanistic analyses have documented the ability of RvD_1_ to shift enzymatic balance within the arachidonic acid pathway, resulting in promoting 5-LOX nuclear exclusion and enhancing LXA_4_ secretion in macrophages [22]. In addition, RvD_2_ has been reported to prevent thrombosis and inhibit pro-inflammatory cytokine expression such as TNF-α and IL-1β in a mouse burn wound model [24]. In the present study, LTD_4_ displayed pro-inflammatory effects in human bronchial rings by increasing the density ratios of 5-LOX and CysLTR1 proteins. These effects were clearly blunted after the addition of nanomolar concentrations of RvD_2_ in the culture media through a decrease in the expression level of 5-LOX and CysLTR1 in LTD_4_-pre-treated human bronchi. This observation is thus well correlated with the mode of action of RvD_1_ observed in IL-13 pre-treated human bronchi in which COX-2 expression was blunted by the tri-hydroxylated DHA derivative [21]. Note that the use of 300 nM RvD_2_ was based on previous data where 300 nM SPM (such as RvD1 or RvE1) blunted the airway and vascular hyperreactivity in *in vitro* models of human bronchi and arteries [21, 26].

TNF-α-mediated activation of p38 MAP kinase and AP-1has been reported to play a key role in the genesis of airway inflammation, mainly through the production of various pro-inflammatory cytokines in chronic respiratory diseases [10, 34, 35]. Moreover, Mukhopadhyay et al. suggested that the up-regulation of cytokines including TNF-α contribute to the development of pulmonary pathophysiology such as asthma and COPD [12]. Accordingly, when the membrane TNF-receptor is stimulated, cytoplasmic p38-MAP kinase is activated. The AP-1 complex is concomitantly formed and translocated into the nucleus where it enhances the phosphorylation of c-Fos and c-Jun subunits promoting the transcription of various genes mainly involved in inflammatory and proliferative processes [11, 34, 35]. Our current data demonstrate the substantial up-regulation in the phosphorylation levels of p38-MAPK, c-Fos and c-Jun of AP-1, as witnessed in TNF-α-treated human bronchi, whereas RvD_2_ conversely blunted these pro-inflammatory signals. These data also suggest that all proteins for which the detection is dependent on the activation of p-38 MAPK and/or AP-1 are likely down-regulated in the presence of RvD_2_. Such findings are in keeping with a previous report demonstrating the blunting of the TNF-α/NFκB pathway by RvD_1_ in IL-13 pre-treated human bronchi [21]. Similarly, MAG-EPA and MAG-DPA were furthermore shown to normalise the NFκB and AP-1 signalling pathways in guinea pig tracheal rings pre-treated with exogenous TNF-α [36, 37]. In addition to corroborating observations in a previous study demonstrating the involvement of RvD_1_ in the inactivation of the 5-lipoxygenase pathway in macrophages [22], our data obtained in LTD_4_-treated human bronchi also reveal that TNF-α mediated activation of the 5-LOX/CysLTR1 pathway is normalised upon RvD_2_ treatments.

LTD_4_ and TNF-α have previously been shown to be enhanced and released in lungs from asthmatic patients [9, 10, 11, 12], with LTD_4_ being a potent broncho-active agent in human ASM [10]. The mechanism by which LTD_4_ contracts the smooth muscle appears to be the result of a complex phenomenon that is principally mediated by CysLT1R (a G protein-coupled receptor) [27, 29]. Previous studies have shown that LTD_4_ is one of the few molecules capable of inducing airway hyperresponsiveness; moreover LTD4-induced tone in human bronchi is inhibited by pranlukast, zafirlukast or pobilukast [4, 29]. Accordingly, a previous report has furthermore demonstrated that LTD_4_, but not LTC_4_, induced a leftward displacement of the concentration-response curve to histamine in bovine ASM strips [8]. In addition, the intrinsic tone in human isolated bronchi was inhibited by montelukast, a specific cysteinyl- leukotriene antagonist [4]. Our current results demonstrate that 48 h LTD_4_ treatment of human bronchial tissue increases the inflammation and AHR and that 300 nM RvD_2_ is able to reverse theses airway inflammatory parameters. 1μM LTD_4_ pre-treated human bronchi are likely to be maximally contracted following the 48 h incubation period. It would have been interesting to demonstrate the potential effects of prolonged LTD_4_-induced contraction under these conditions. As a matter of fact, Plate 1of the supplementary material, demonstrates that 1μM LTD_4_ fully induce the expression of CysLTR1. Recent findings have demonstrated that AHR developed in tissue culture models is mainly triggered by an inflammatory process mediated by the TNF-α cytokine [11, 19, 37]. Accordingly, Morin et al. have previously reported that spasmogens cause an increase in tone in TNF-α-treated bronchi whereas a neutralising TNF-α antibody (Infliximab) or an NFκB inhibitor consistently reduced the Ca^2+^ sensitivity of TNF-α pretreated bronchial myofilaments [11]. In the present study, RvD_2_ was found to abolish the hyperresponsiveness induced by short-term (48 h) LTD_4_ or TNF-α pre-treatment of human bronchial explants. Indeed, in the presence of nanomolar concentrations of RvD_2_, the contractile responses to various bronchoactive agents were reset to the level recorded under control conditions (cultured and untreated human bronchi). The direct link between inflammatory conditions and hyperresponsiveness in human bronchi, while often suggested, has only been sparsely assessed. Our data attest that inflammation can lead to increased mechanical reactivity and that RvD_2_ would prevent the abnormal pharmacological reactivity in human bronchi.

RvD_2_ and lipoxin A_4_ share a significant structural homology (including three hydroxyl groups and four double bounds), which are features of several resolving compounds [16]. In normal and tumoural mammary tissues, enzymes involved in lipoxin A_4_ and RvD_2_ synthesis are expressed, with these lipid mediators also reported to display a crucial role in oestrogen-dependent breast cancer progression [38]. In human bronchial explants, it has been demonstrated that LXA_4_ mediates beneficial effects via formyl-peptide receptor 2 signalling [39]. This observation is correlated with the ability of LXA_4_ to significantly inhibit the platelet-activating factor-induced increases in leukocyte-platelet aggregates and TNF-α-triggered AHR [39]. Herein, we also demonstrate that the ALX/FPR2 receptor is expressed in human bronchi in normal and pro-inflammatory conditions and that the pro-resolving effects triggered by RvD_2_ are partially mediated by ALX/FPR2 receptors. Although LC-MSMS is the gold-standard for SPM measurement, it would be interesting to measure the LXA_4_ levels in MAG-DHA (RvD2 precursor) or RvD2 pre-treated human bronchi. Regarding both inflammation and AHR properties, our current data demonstrate that RvD_2_ displays coherent resolving effects that are partially antagonised by the use of an ALX/FPR2 receptor antagonist. Accordingly, 1 μM WRW4 abolished the beneficial effects of RvD_2_ on TNF-α-induced activation of the 5-LOX/CysLTR1 pathway as well as TNF-α-induced airway hyperresponsiveness. Recently, Chiang et al. reported on a novel RvD_2_–GPR-18 resolution axis in which RvD_2_, acting through GPR-18 membrane receptor, stimulated human and mouse phagocyte functions, controlled bacterial infections and promoted organ protection [40]. Their study also showed that RvD_2_ reversed both *E*. *coli* and *Staphylococcus aureus* infections by neutralising PMN infiltration and accelerating phagocyte clearance of bacteria. In addition, it has been demonstrated that all RvD_2_ beneficial effects are lost in GPR18-deficient mice [40]. In keeping with these observations, our data clearly demonstrate that, under stringent pro-inflammatory conditions, D-series resolvins are instrumental pro-resolving agents, which are consistently and concomitantly antagonised by the ALX/FPR2 blocking peptide.

Work by others [40] show that deficiency of the RvD2 receptor leads to decreased levels of RvD2 and other 15-lipoxygenase products indicating a possible regulation of 15-LOX by RvD2. Thus It would be valuable to examine 15-LOX regulation by RvD2 in the airways. In contrast, Morin et al., have demonstrated that MAG-DHA and RvD1 mediated potent anti-inflammatory effects and that the combination of MAG-DHA + 15-LOX and 5-LOX inhibitors reversed the anti-inflammatory effects induced by MAG-DHA in TNF-pretreated human bronchi [19].

In summary, we report the first evidence that RvD_2_ prevents inflammatory responses in a human model of airway hyperresponsiveness triggered by LTD_4_ or TNF-α. According to our experimental results following inflammatory stimulus, this bioactive lipid mediator blunts the LTD_4_-mediated activation of the lipoxygenase–Cysteinyl- leukotrienes receptor pathway and curbs the enhanced phosphorylation of p38-MAPK and AP-1 subunits induced by TNF-α which, in turn, inhibits the resulting hyperresponsiveness. Concomitant exogenous addition of an ALX/FPR2 peptide blocker also reduced the antiphlogistic and broncho-modulatory effects of RvD_2_. Taken together, these data provide new insights into the pro-resolving properties of RvD_2_ in human smooth muscle cells. These findings may potentially represent new prospective and protective clinical targets in countering inflammatory and hyperreactivity components in chronic airway diseases.

## Supporting Information

S1 Plate (TIF)

## Abbreviations

AHR: Airway hyperresponsiveness
AP-1: Activator protein-1
ASM: Airway smooth muscle
CysLTR1: Cysteinyl leucotriene receptor 1
HB: Human bronchi
LTD_4_: 5S-hydroxy-6R-(S-cysteinylglycinyl)-7E,9E,11Z,14Z-eicosatetraenoic acid
RvD_2_: 17S,8R,17S-trihydroxy-4Z,9E,11E,13Z,15E,19Z-docosahexaenoicacid
TNF-α: Tumour necrosis factor alpha
