# Supplementary figures and images for: Pro-Resolving Effects of Resolvin D2 in LTD4 and TNF-α Pre-Treated Human Bronchi
Source: PLoS One. 2016 Dec 9;11(12):e0167058. doi: 10.1371/journal.pone.0167058 (PMC5148597; doi:10.1371/journal.pone.0167058)

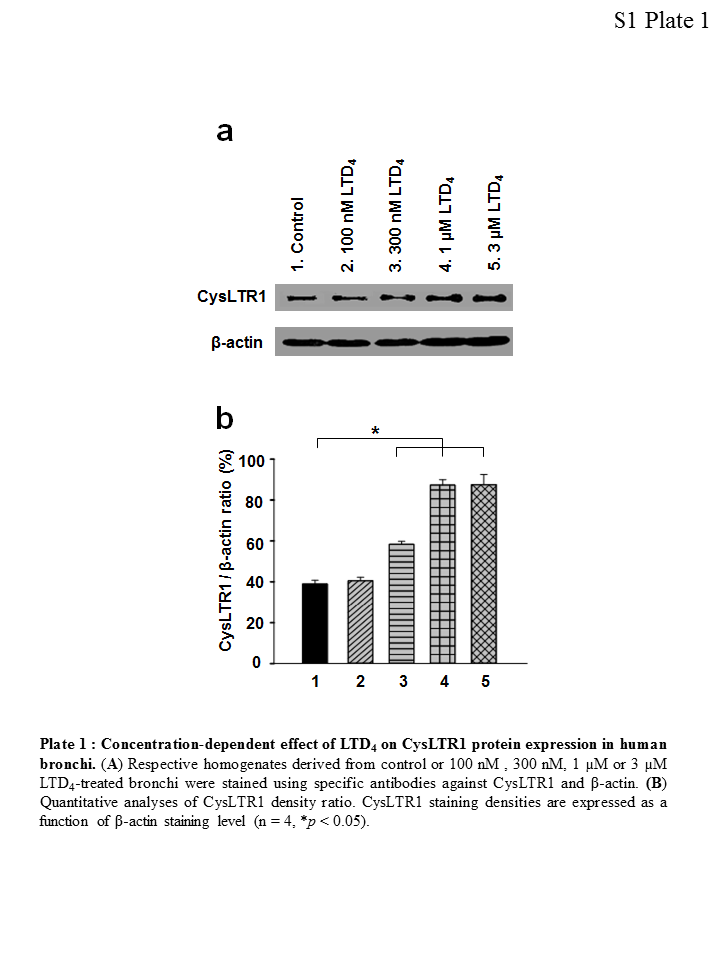

Supplement: S1 Plate — (TIF) [file pone.0167058.s001.tif]
